# Phase controlled synthesis of bifunctional TiO_2_ nanocrystallites *via*d-mannitol for dye-sensitized solar cells and heterogeneous catalysis†

**DOI:** 10.1039/d0ra01366h

**Published:** 2020-04-14

**Authors:** Abdullah M. Al-Enizi, T. A. J. Siddiqui, Shoyebmohamad F. Shaikh, Mohd Ubaidullah, Ayman Yousef, Rajaram S. Mane, Abu ul Hassan Sarwar Rana

**Affiliations:** Department of Chemistry, College of Science, King Saud University Riyadh 11451 Saudi Arabia mtayyab@ksu.sa; Swami Ramanand Teerth Marathwada University Nanded-431606 MS India rajarammane1970@srtmun.ac.in; Department of Mathematics and Physics Engineering, Faculty of Engineering at Mataria, Helwan University Cairo 11718 Egypt; Department of Intelligent Mechatronics Engineering, Sejong University Seoul South Korea rana@sejong.ac.kr

## Abstract

The crystal architecture of TiO_2_ was successfully tailored *via* a low-temperature (≤200 °C) hydrothermal process in the presence of d-mannitol for feasible applications in dye-sensitized solar cells (DSSCs) and heterogeneous catalysis. In the development of anatase-TiO_2_ (A-TiO_2_), d-mannitol does not merely acts as a complexing agent to manage the zigzag chains of octahedral TiO_6_^2−^ with dominant edge sharing but also performs as a capping agent by influencing the hydrolysis process during nucleation, as confirmed by Fourier-transform infrared spectroscopy and dynamic light scattering studies. After physical measurements, the as-synthesized nanocrystallites (NCs) of A-TiO_2_ were used in DSSCs, where a fascinating power conversion efficiency (PCE) of 6.0% was obtained, which showed excellent performance compared with commercial anatase-TiO_2_ (CA-TiO_2_: 5.7%) and rutile-TiO_2_ (R-TiO_2_) obtained without d-mannitol (3.7%). Moreover, a smart approach was developed *via* the A-TiO_2_ catalyst to synthesize pharmaceutically important C-3 alkylated 4-hydroxycoumarins through different activated secondary alcohols under solvent-free, and heat/visible light conditions. In addition, the catalytic activity of the so-produced A-TiO_2_ catalyst under solvent-free conditions exhibited remarkable recyclability with up to five consecutive runs with negligible reduction, which is superior to existing reports, and clearly reveals the novelty, and green, sustainable nature of the as-synthesized A-TiO_2_ catalyst. A plausible reaction mechanism of both coupling partners was activated through the interaction with the A-TiO_2_ catalyst to produce valuable C-3 alkylated 4-hydroxycoumarins with 95% yield and high selectivity.

## Abstract 2

The crystal architecture of TiO_2_ was successfully tailored *via* a low-temperature (≤200 °C) hydrothermal process in the presence of d-mannitol for feasible applications in dye-sensitized solar cells (DSSCs) and heterogeneous catalysis.

## Graphical abstract

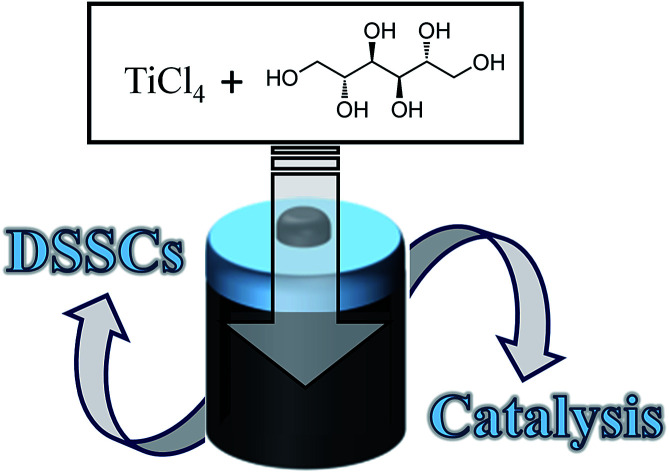

## Introduction

Titanium dioxide (TiO_2_) mainly exists in three polymorphs: anatase (tetragonal), *i.e.*, A-TiO_2_; brookite (orthorhombic), and rutile (tetragonal), *i.e.*, R-TiO_2_; these are regarded as different orientations of the shared edges/corners of the [TiO_6_^2−^] octahedra.^1^ The TiO_2_ polymorphs play an important role due to their outstanding electrical, optical, and catalytic properties, as well as their chemical stability under both acidic and alkaline conditions, which have enabled their applications in energy storage, photovoltaic cells, and photocatalysis in the last few decades.^2^ For instance, A-TiO_2_ is largely preferred in photovoltaics and photocatalysis applications over the other two polymorphs, due to its abundance, environmental friendliness, low cost, excellent electron mobility, catalytic activity, and availability of several cleanroom-free chemical/physical synthesis methods. Generally, the higher performance of the A-TiO_2_ over R-TiO_2_ because of its maximum dye-loading capacity and superior incident photocurrent conversion efficiency. Moreover, the high refractive index and UV absorptivity of R-TiO_2_ make it a potential candidate in the application of optical communication devices.^3^ The crystal phase transition of TiO_2_ polymorphs using simple, cost-effective, and low-temperature solution-based methods with different morphologies has attracted considerable research interest for technologically important and practical applications. Normally, peptization, a modifier/surfactant/chelating agent, capping agent, cohesive agent, and annealing temperature play crucial roles during the phase transition pathways of these TiO_2_ polymorphs, in addition to intrinsic parameters for instance pore size, concentration of the Ti precursor, energy on the surface, aggregation rate, pH value of solution, and crystal growth dynamics.^4,5^ Many solution-based methods have been reported for the synthesis of different TiO_2_ polymorphs such as solvothermal, wet chemical, and sol–gel.^6^ Among these, hydrothermal synthesis is used to obtain phase homogeneity and morphological control at low temperatures.^7^ Zhou *et al.* reported the hydrothermal crystallization process for the synthesis of TiO_2_*via* a dissolution–crystallization mechanism.^8^ At a specific temperature, hydrated Ti ions begin to dissolve to produce Ti^4+^ hydrated octahedral monomers, which, eventually, form polymers *via* a condensation reaction. Consequently, connection with octahedral monomers, the formation and growth of the TiO_2_ nuclei are initiated.^9^ Thus, the architecture of Ti^4+^ hydrated octahedral monomer and nucleation process under hydrothermal conditions are major components involved in the fabrication of TiO_2_ polymorphs. Recently, Oskam *et al.* reported the nucleation and growth mechanism of different TiO_2_ polymorphs by applying precursor chemistry, which was mainly dependent on the utilization of reactants.^10^ Joo *et al.* studied the crystal structures of TiO_2_ polymorphs that are controlled from rutile to anatase without and with d-sorbitol acting as a complexing agent.^11^ Yanqing *et al.* examined the impact of the TiCl_4_ concentration and pH of solution on nucleation processes of TiO_2_ polymorph.^12^ Until now, the formation of TiO_2_ polymorphs with a definite crystal structure, shape, surface orientation, and nucleation process under hydrothermal conditions remain uncharted; thus, the correlation between crystal structure formation, and the hydrolysis ratio needs to be investigated for various applications.^13^

In the present work, we successfully synthesized pure phase A-TiO_2_ NCs *via* a facile, low-temperature, and economical hydrothermal method in the presence of d-mannitol, which play role of both capping as well as complexing agent. The d-mannitol was selected due to its non-toxic biological origin, environmental friendliness, low-cost, capping ability, and capability to form an intermediate complex. The possible reaction mechanism to the formation of A-TiO_2_ is based on the different orientation of shared corners and edges of the TiO_6_^2−^ octahedral. The nucleation process of TiO_2_ has been derived by [Ti(OH)_*x*_(d-mannitol)_*y*_Cl_*z*_]^*n*−^, where, 4 ≤ *x* + *y* + *z* ≤ 6 during the hydrolysis process, and the relationship between the crystal structure formation and hydrolysis process has been explored. Furthermore, for comparison, we confirmed that the reactions that proceed without d-mannitol result in a phase of pure R-TiO_2_ rather than A-TiO_2_. The effect of the hydrothermal reaction conditions on the crystal architecture formation as well as the photovoltaic and photocatalytic properties was investigated, where A-TiO_2_, synthesized using d-mannitol, exhibited an enhanced DSSCs performance compared to that of the R-TiO_2_ prepared without it. The presented method for obtaining both A-TiO_2_ and R-TiO_2_ polymorphs is facile, environmentally friendly, exhibits long-term chemical stability, and involves a single-step. The TiO_2_ polymorphs synthesized through this method are used in various applications, including in DSSCs and heterogeneous catalysis.

## Experimental details

### Chemicals

AR grade chemicals were utilized without any refinement. Titanium(iv) chloride (TiCl_4_, 99.9%), d-mannitol (>98%), ethanol (96%), and α-terpineol (90%) were purchase from Sigma Aldrich company. Acetonitrile (99.8%), *tert*-butanol (99.5%), and ethyl cellulose were purchased from Alfa Aesar. The N-719 dye (Ruthenium-535, bis TBA) and iodide electrolyte (Iodolyte AN-50), were commercially available and used as received.

### Hydrothermal synthesis of A- and R-TiO_2_

**Scheme 1:**
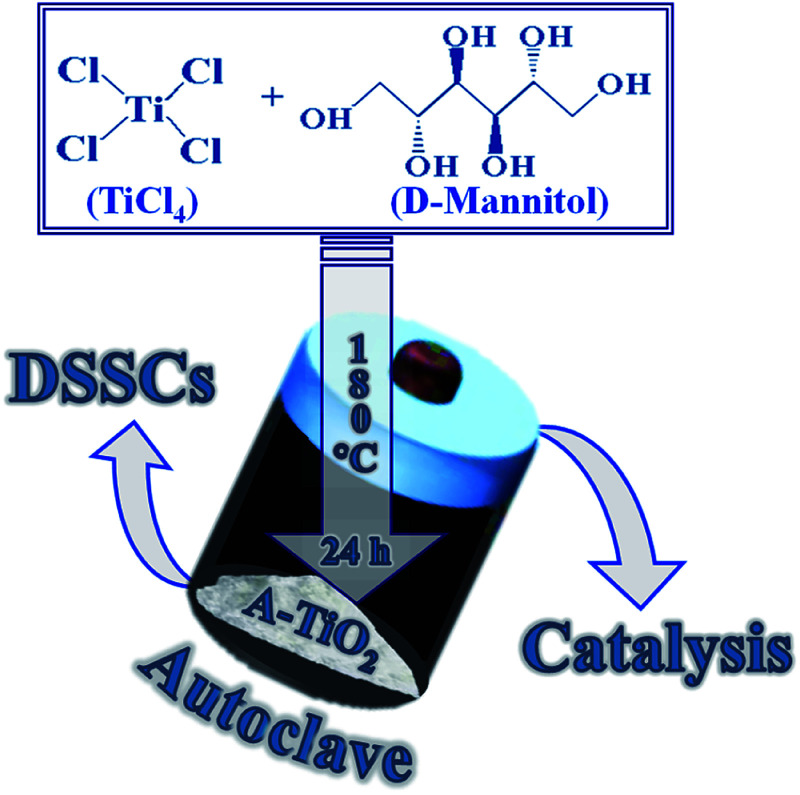
The hydrothermal synthesis process of A-TiO_2_ in presence of d-mannitol for DSSCs and heterogeneous catalysis applications.

The NCs of A- and R-TiO_2_ polymorphs were produced by a simple and elegant one-step hydrothermal route. Typically, for the A-TiO_2_ NCs, 1 M TiCl_4_ and 2.5 g of d-mannitol were dissolved in DI-water water (DI, Milli-Q water; 18.2 MΩ cm). The reaction mixture was transferred to high pressure Teflon reactor and kept at constant temperature of 180 °C for 24 h. The as-procured yellowish-white precipitate was separated on centrifugation at 4000 rpm for 10 min, followed air annealing at 550 °C for 30 min to get white powder of A-TiO_2_NCs (Scheme 1). A similar experimental condition was applied for the synthesis of R-TiO_2_NCs, except there was no d-mannitol in the precursor solution. A 100% A-TiO_2_ paste of ∼20 nm sized NCs, named CA-TiO_2_, was purchased from ENB Korea and used without any modification as the reference.

### Fabrication of the DSSCs

A fluorine-tin-oxide substrate (FTO, TEC15, Pilkington, 15 ohm/sq.) was sonicated through detergent in DI-water, acetone, and isopropanol for 20 min, in sequence, and dried under nitrogen gas flow, followed by 15 min ultraviolet–ozone treatment to remove presence of any unwanted residues. Typically, the TiO_2_ paste was made by mixing of 1.0 g of TiO_2_ NCs, 2.0 g of α-terpineol, and 0.3 g of ethyl cellulose in 5.0 mL of ethanol with continuous stirring to obtain homogeneous paste for 6 h. Subsequently, the as-prepared NCs of A-, R-, and CA-TiO_2_ pastes were coated on the FTO substrate *via* a simple doctor-blade method using an adhesive tape on both side as a spacer. About eight-ten-micron thickness TiO_2_ films for each case were prepared and annealed at 550 °C in a muffle furnace under an air environment at a constant heating 5° min^−1^ rate for 30 min. For sensitization, these TiO_2_ photoanodes were heated to 60 °C and kept for adsorbing 0.3 mM N-719 dye solution of 1 : 1 *tert*-butanol : acetonitrile for 24 h at 27 °C in the dark. The Pt counter electrode having mirror finish, deposited by DC magnetron sputtering on the conducting FTO substrate, was used as the counter electrode. The two electrodes, *i.e.*, TiO_2_ with N-719 dye and Pt were clipped together, and to prevent the leakage of the iodine electrolyte solution heat resistant sublimation tape was used as a sealant. The purpose of mask is to allow light to fall only onto a active area of all the devices, *i.e.* the area was 0.5 cm^2^ defined by the mask.

### Catalyst activity

A combination of 4-hydroxycoumarin (1 mmol) and 4-methyl-phenylethanol (1 mmol) and A-TiO_2_ NCs powder (20 mg) was stirred at 70 °C for 5 h. After completion of the reaction (monitored by TLC) ethyl acetate was added to the reaction mixture and the catalyst was collected by filtration. The organic solvent was concentrated under reduced pressure. After purification by recrystallization with hot ethanol, C-3 alkylated 4-hydroxycoumarins were obtained. Moreover, to achieve the best reaction conditions, the effects of various amount of catalyst, reaction time, optimal temperature, and solvent were meticulously investigated. A model reaction between 4-hydroxycoumarin and activated alcohols was monitored to ascertain the optimal reaction parameters. After completing reaction, obtained products were confirm and characterized by ^1^H NMR spectral data.

### Measurements and characterizations

The morphology observation of A-TiO_2_ and R-TiO_2_ were confirmed from the surface digital images by field emission scanning electron microscopy (FE-SEM, FEI Nova NanoSEM). High-resolution transmission electron microscopy and selected area electron diffraction (SAED) measurements analyses were conducted by (HR-TEM, FEI TECNAI G2 20 S-TWIN) by using a 200 kV accelerating voltage. The phases of the TiO_2_ NCs were validated by powder X-ray diffraction patterns (Shimadzu, XRD-6000, Japan) obtained using *λ* = 0.1542 nm. The phase was confirmed by Raman microscopy (Renishaw, inVia Raman microscope, UK) also. The laser beam (*λ* = 532 nm) was focused using a lens to produce a spot on the TiO_2_ film surfaces. The Fourier-transform infrared (FTIR) spectroscopy spectra were obtained to confirm intermediate reaction state from 500 to 4000 cm^−1^ using Nicolet, iS10, Smart MIRacle, Thermo Scientific. X-ray photoelectron spectroscopy (XPS) spectra were acquired using a PHI 5000 VersaProbe (Ulvac-PHI) to confirm the surface elements and the chemical states. The binding energies were used for internal calibration of C 1s peak (284.6 eV). The specific surface areas of A-TiO_2_ and R-TiO_2_ powders were measured through Brunauer–Emmett–Teller spectra on nitrogen physisorption instrument (BET, Belsorp II, BEL Japan INC). The amount of dye adsorbed over A-, R-, and CA-TiO_2_ photoelectrode was confirmed by desorbing in a 0.1 M NaOH water–ethanol (v/v = 1 : 1) mixed solution, followed by UV-Vis spectrophotometer (Varian Cary 5000 spectrophotometer) measurement by UV-Vis spectrophotometer (Shimadzu UV-2450) at 540 nm. Simulated sunlight provided by a solar simulator (Sun 2000 ABET 5 Technologies, USA) fitted with an A.M. 1.5G filter was used, and the intensity of one sun (100 mW cm^−2^) was calibrated on standard silicon solar cell. A Keithley 2400 source meter was used to estimate the current density–applied voltage (*J*–*V*) spectra of the A-, R-, and CA-TiO_2_ photoanodes. The computer-controlled electrochemical impedance spectroscopy (EIS) measurement spectra were recorded on IviumStat Technologies, Netherland at a bias voltage of 0.70 V in a frequency range of 150 kHz to 0.1 Hz under dark condition.

### NMR spectral data

#### (1) 4-(1-*p*-Tolylethoxy)-2*H*-chromen-2-one (3a)

1H (400 MHz; CdCl_3_-*d*_6_): *δ* 1.59–1.63 (d, 3H, *J* = 16 Hz), 2.33 (s, 3H), 4.67–4.71 (q, 1H, *J* = 16 Hz), 6.04 (S, 1H), 7.22–7.63 (m, 8H).

#### (2) 3-(1-(4-Bromophenyl)ethyl)-4-hydroxy-2*H*-chromen-2-one (3b)

1H (400 MHz; CdCl_3_-*d*_6_): *δ* 1.653–1.671 (d, 3H, *J* = 0.), 4.696–4.750 (q, 1H, *J* = 7.2 Hz, 14.4 Hz), 6.33 (s, 1H), 7.198–7.676 (m, 8H).

#### (3) 3-(1-(4-Chlorophenyl)ethyl)-4-hydroxy-2*H*-chromen-2-one (3c)

1H (400 MHz; CdCl_3_-*d*_6_): *δ* 1.65–1.69 (d, 3H, *J* = 16 Hz), 4.65–4.75 (q, 1H, *J* = 12 Hz and *J* = 28 Hz), 6.33 (bs, 1H), 7.22–7.72 (m, 8H).

#### (4) 4-(1-(2,4-Dichlorophenyl)ethoxy)-2*H*-chromen-2-one (3d)

1H (400 MHz; CdCl_3_-*d*_6_): *δ* 1.65–1.69 (d, 3H, *J* = 16 Hz), 4.65–4.72 (q, 1H, *J* = 28 Hz), 7.11–7.69 (m, 9H), 6.33 (s, 1H).

## Results and discussion

### Reaction mechanism

**Scheme 2:**
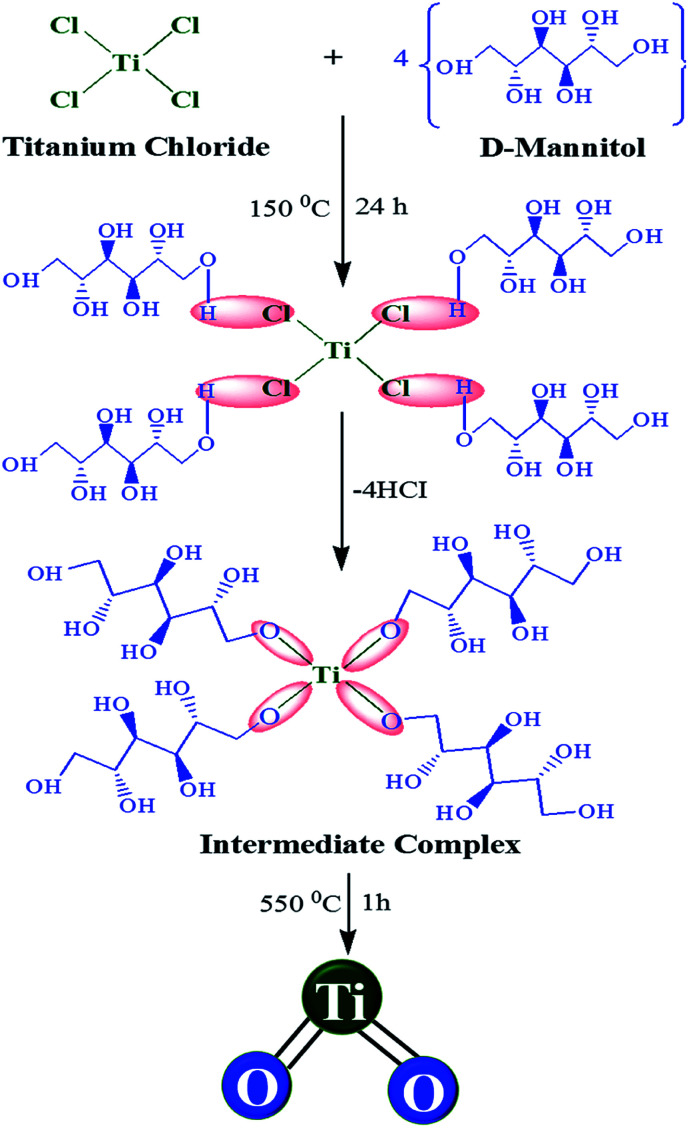
Plausible reaction mechanism to the formation of A-TiO_2_ NCs.

The TiO_6_^2−^ octahedral with different orientations of shared edges and corners are responsible for affording A- and R-TiO_2_. Basically, R-TiO_2_ is connected by ten octahedra, in which eight are corner-shared and two are edge-shared; the crystal nuclei for rutile TiO_2_ is made up of linear chains of TiO_6_^2−^ octahedra connected by corner sharing.^14^ In contrast, A-TiO_2_ is connected by eight octahedra, in which four are corner-shared and four are edge-shared, making zigzag chains of TiO_6_^2−^ octahedra that are linked to each other through edge-sharing bonding.^15^ The density of A-TiO_2_ (3.84 g cm^−3^) less than R-TiO_2_ (4.26 cm^−3^) due to more edge-shared, and larger interstitial spaces between the octahedra.^16^ Notably, the crystal structure of TiO_2_ can be agglomerated through different edge-shared and corner-shared TiO_6_^2−^ octahedra orientations during the condensation processes. Generally, when Ti precursor reacts with water molecules, the coordination number of Ti^4+^ increases from four to six, owing to the acceptance of oxygen lone pairs from nucleophilic ligands through its vacant d-orbitals. These six-fold octahedra structural units can be combined into the final crystal structure formation through condensation.^17^Scheme 2 has illustrated the formation of A-TiO_2_ reaction mechanism. It was found that d-mannitol anions can be substituted for Cl anions during the hydrolysis process to form [Ti(OH)_*x*_(d-mannitol)_*y*_Cl_*z*_]^*n*−^ intermediate complexes during crystallization. Similarly, Chen *et al.* demonstrated that BiOCl surface selectively adsorb molecules of d-mannitol to from hydrogen bonds with exposed oxygen atoms to decrease the surface energy during the crystal growth formation.^18^ Effect of d-mannitol on the crystal structure formation and nucleation process of A-TiO_2_ is worth for future investigations. In addition, the hydrochloric acid present in the TiCl_4_ aqueous solution could act as a catalyst not only facilitating the formation of crystal growth *via* condensing the intermediate complex but also nucleation process and related species.^19^

### Intermediate complexes

To confirm the formation of intermediate complexes, the FTIR spectrum was investigated. On completing hydrothermal reaction between TiCl_4_ and d-mannitol, the white precipitate of intermediate complex was transferred into a centrifuge tube at 8000 rpm for 10 min, and washed many times with DI-water, and subsequently dried at room temperature before FTIR measurement. Fig. S1a in ESI† shows the FTIR spectrum of standard d-mannitol consisting of strong –OH and –C–O stretching vibration peaks at 3366, 1080, and 1015 cm^−1^, as well as the peaks indicative of the C–H stretching vibration at 2983, 2947, and 2901 cm^−1^. The peak indicative of –CH_2_ scissor vibrations is noted at 1417 cm^−1^.^20^ Fig. S1b† shows the FTIR spectrum of the intermediate complex between Ti^4+^ and d-mannitol ions, where the molecular interaction of d-mannitol ions with Ti ions, in addition to overlapping, is shifted positively and negatively, thereby validating the construction of a complex structure. The FTIR spectrum of the intermediate complex showed a broad peak at 3167 cm^−1^, which coincides to –OH stretching vibrations. A broad peak of the –OH stretching vibrations validated the presence of hydroxyl groups over different sites. The high-intensity FTIR peak near 600 cm^−1^ could be assinged to TiO_2_.^21^

### Hydrolysis rate estimation

**Fig. 1:**
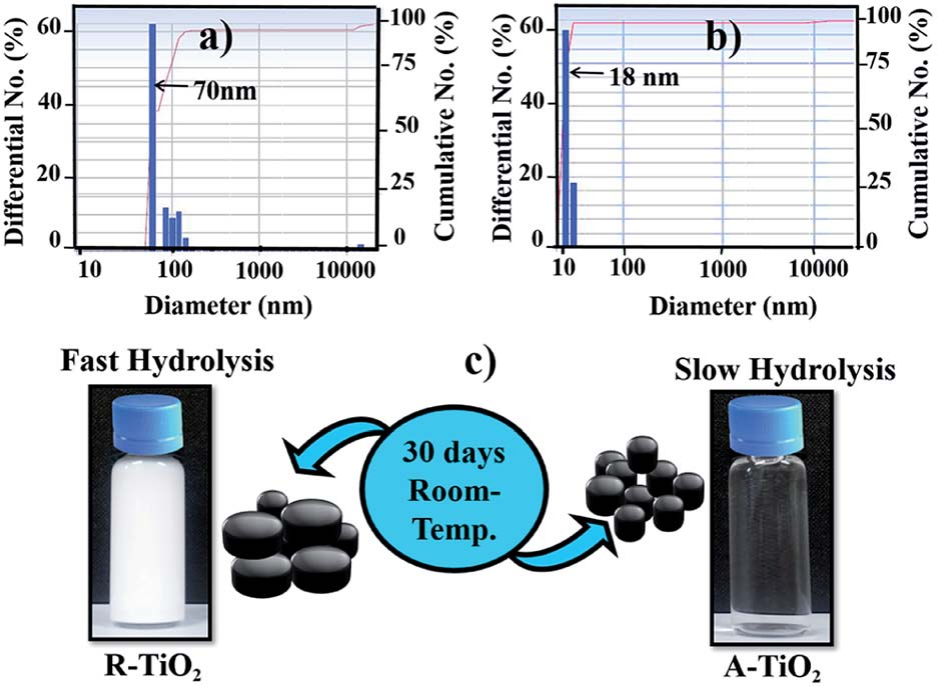
DLS spectra of; (a) 1 M TiCl_4_, (b) TiCl_4_-0.1 M d-mannitol in aqueous solution, and (c) diagram showing 30 days stability at a room-temperature where hydrolysis takes place in aqueous solution of TiCl_4_ without d-mannitol (R-TiO_2_) and TiCl_4_-0.1 M d-mannitol (A-TiO_2_).

The effect of d-mannitol (0.1 M) on the hydrolysis process of TiCl_4_ (1 M) was measured through dynamic light scattering technique. As shown in Fig. 1a, in the presence of d-mannitol, TiCl_4_ in aqueous medium under goes hydrolysis to develop slow particle agglomeration with a small size distribution of ∼18 nm. TiCl_4_ in aqueous solution, in the absence of d-mannitol undergoes hydrolysis to form fast particles agglomeration with ∼70 nm size distribution (Fig. 1a), confirming the interaction of d-mannitol with TiCl_4_ that prevents the rapid hydrolysis (Fig. 1b). The coordination of d-mannitol anions with TiCl_4_ inhibit the crystal growth resulting the formation of small-sized A-TiO_2_ NCs. For an improved understanding of the hydrolysis reaction, we maintained both samples for more than thirty days (TiCl_4_ and TiCl_4_-0.1 M d-mannitol) at room temperature (Fig. 1c). After two days, aqueous medium of TiCl_4_ solution changed to white coloured precipitate with sediments. This white coloured precipitate subsequently agglomerated, and settle down slowly on the surface of bottom. However, the TiCl_4_-0.1 M d-mannitol solution remained transparent without precipitated even keep at one month, which is also supported by the dynamic light scattering method, where to preventing the fast hydrolysis process Ti cations could bond with d-mannitol anion at room temperature. We assumed that the slow hydrolysis process taking place between TiCl_4_-0.1 M d-mannitol solutions plays important role in the formation of small particles size, which ultimately supports the phase transformation process, from rutile to anatase. Though, to initiate the nucleation process during the hydrolysis of A-TiO_2_NCs from the d-mannitol comprising solution, suitable temperature is required. Xiong *et al.* reported, in their BiOCl study, that d-mannitol not only behaved as a cohesive agent to prompt the assembly of the structural morphology but also acted as a capping agent to prevent the crystal anisotropic growth.^22^ To validate the comparison, the acidity (pH = 0.6) of both solutions were kept the same for the dynamic light scattering measurement.

### Structural elucidation

**Fig. 2:**
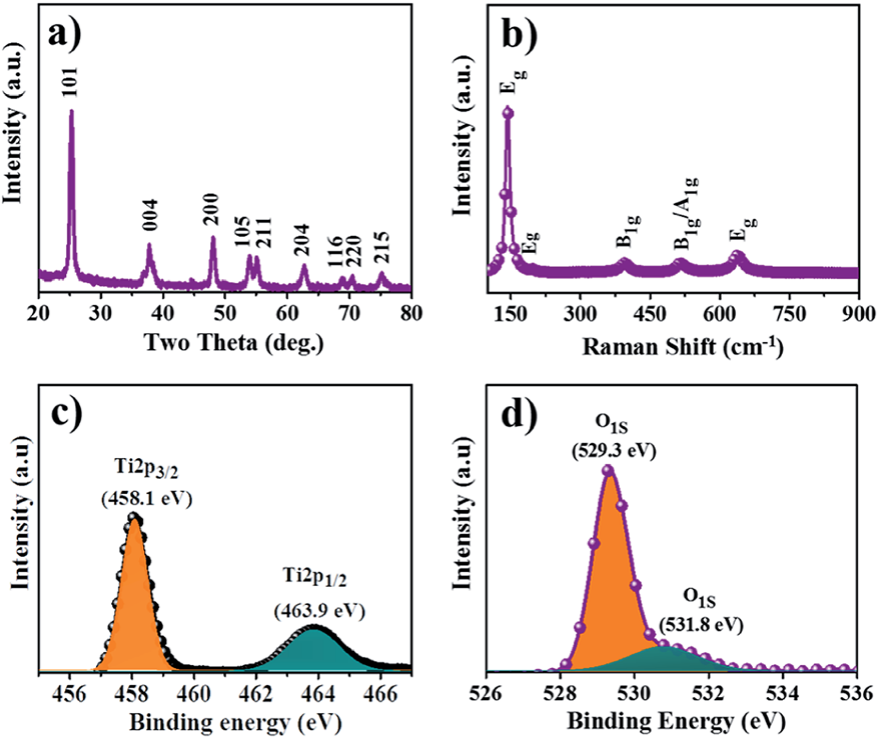
(a) X-ray diffraction, (b) Raman spectrum, (c) XPS analysis of Ti_2p_, and (d) O_1S_ of A-TiO_2_ NCs with d-mannitol.

XRD patterns were used to characterize the crystallite structures, phases, and average sizes of the as-obtained A- and R-TiO_2_. The XRD trends of the as-synthesized TiO_2_ NCs with and without d-mannitol are respectively shown in Fig. 2a and S2a.† The prior XRD pattern confirmed peaks at 25.3°, 37.8°, 48.0°, 53.8°, 55.0°, 62.6°, and 68.8°, which are ascribed to the 101, 004, 200, 105, 211, 204, and 116 reflection planes of A-TiO_2_ (JCPDS no. 21-1272),^23^ respectively, whereas in later XRD pattern, the peaks at 27.4°, 36.0°, 39.1°, 41.2°, 44.0°, 54.3°, 56.6°, 62.7°, 64.0°, 69.7°, and 72.4° in association with (110), (101), (200), (111), (210), (211), (220), (002), (310), (112), and (311) for R-TiO_2_ (JCPDS data file no. 21-1276) are respectively evidenced.^24^ There were no difficulties in separating the A and R phases of TiO_2_; as for anatase, the first two-theta peak was at 25.3° for *d*_101_, and for rutile, it was at 27.4° for *d*_110_. From the XRD patterns it was clear that, though both A- and R-TiO_2_ NCs were synthesized by the same chemical method, different phases were obtained just by adding d-mannitol. The average crystal size was calculated using the Debye–Scherrer equation. For the as-synthesized A-, CA-, and R-TiO_2_, the average crystallite sizes were 12.0, 16.2, and 19.5 nm, respectively. Raman spectroscopy is an effective tool for identifying the molecules in A-TiO_2_ NCs obtained from d-mannitol. In Fig. 2b, the major Raman shift peaks at 144, 199, 400, 519, and 635 cm^−1^ were attributed to the E_g_, E_g_, B_1g_ (B_1g_/A_1g_), and E_g_ symmetries of A-TiO_2_.^25^ In the Raman spectrum, no additional peaks, except distinctive vibrations, were found, which confirms the formation of pure phase A-TiO_2_. The Raman spectrum demonstrated at 142, 235, 443, and 610 cm^−1^ are attributed to the B_1g_, two-phonon scattering, E_g_, and A_1g_ modes of the rutile phaseof TiO_2_ NCs, respectively (Fig. S2b†). The spin-orbital splitting of Ti 2p_1/2_ and Ti 2p_3/2_ photoelectrons corresponds to a doublet situated at binding energies of 463.9 and 458.1 eV, respectively, as shown in Fig. 2c. The peak separation of Ti 2p_1/2_ and Ti 2p_3/2_ was 5.8 eV, confirming the Ti^4+^ chemical state which is in magnificent consensus with the literature data for A-TiO_2_,.^26^ The O_1S_ singlet of oxygen at 529.3 eV is shown in Fig. 2d. The Lorentzian–Gaussian mix function value was employed in the curve resolution of the individual O_1S_ peaks in the two spectra. The curve resolved O_1S_ signal revealed small sized secondary peak that is positioned at a binding energy of 531.86 eV for –OH species present on the surface.^27^

### Surface morphology evolution

**Fig. 3:**
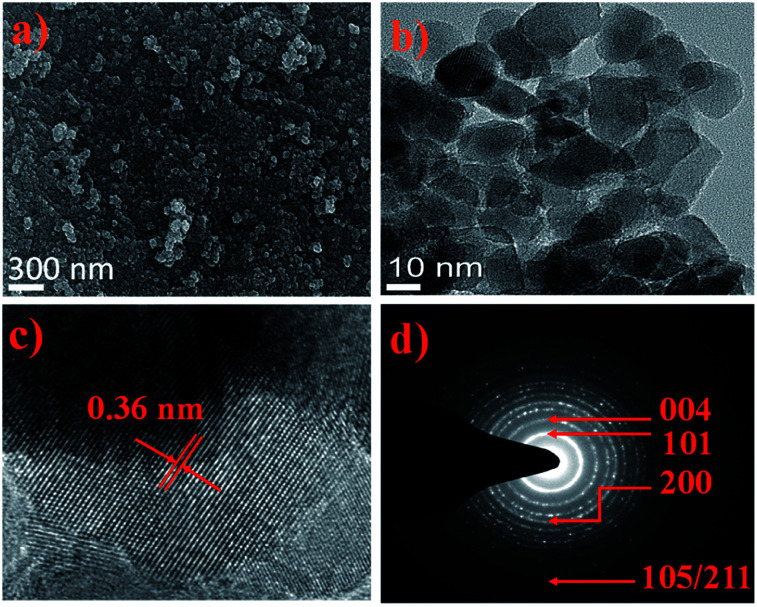
(a) FE-SEM, (b) EDX mapping for Ti and O. (c) HR-TEM, and (d) SAED pattern of A-TiO_2_ NCs.

The surface morphology evolution of A-TiO_2_ NCs is shown in Fig. 3a. The FE-SEM images of A-TiO_2_ confirmed the formation of small-sized spheres, where all the grains are well-interlinked to one another, which would offer various facile transporting channels for electrons (or charge carriers) through the TiO_2_ NCs. The FE-SEM images demonstrate the well-interconnected ∼60 nm average diameter of nanocrystallites, when d-mannitol was absent in the precursor solution as shown in Fig. S2c.† The elemental stoichiometry determined by EDX mapping (Fig. S3†) has evidenced 30.8 and 69.1% atomic proportions for Ti and O, respectively, suggesting the formation of TiO_2_. The HR-TEM results indicate the high crystallinity of TiO_2_ interconnected NCs of about 15 nm in sizes (Fig. 3b). The 0.36 nm interplanar distance in compliance with the (101) plane of A-TiO_2_ NCs (Fig. 3c), and 0.32 nm interplanar distance in compliance with the (110) plane of R-TiO_2_ NCs (Fig. S2d†) were approved.^28^ The (101) facet of the A-TiO_2_ NCs has a relatively low surface energy; thus, it has better stability than those of other facets, and the HR-TEM image also has evidenced clear and sharp rings in reference to the (101), (004), (200), (105), and (211) lattice planes of A-TiO_2_ in the SAED pattern (Fig. 3d).

### Specific surface area and pore-size distribution measurements

**Fig. 4:**
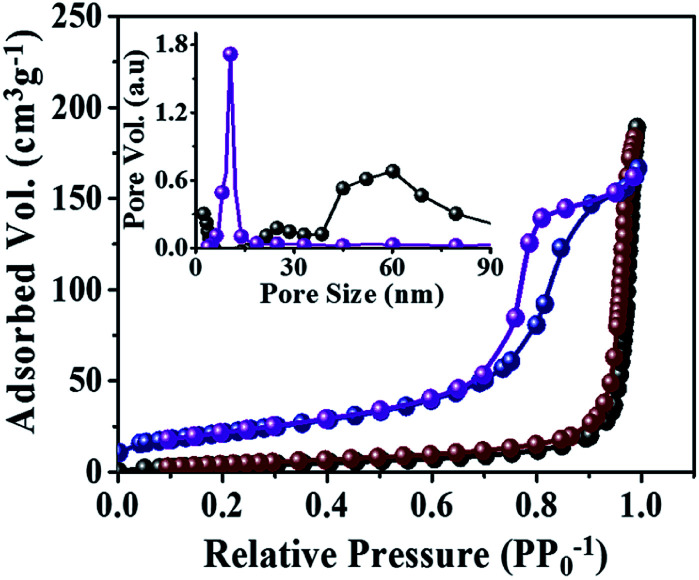
N_2_ adsorption desorption isotherms with inset depicts the pore size distribution of R-TiO_2_ (black and brown dot), and A-TiO_2_ (blue and purple dot), NCs in the presence of d-mannitol. The black and blue dot denoted adsorption and brown and purple dot denoted the desorption of R- and A-TiO_2_ NCs.

The specific surface area and porosity of both R-TiO_2_ and A-TiO_2_ NCs were investigated from the BET and BJH spectra. A type-IV isotherm (Brunauer–Deming–Deming–Teller classification) was due to their mesoporous nature. The hysteresis loop of type H1 suggested that the porosity of A-TiO_2_ is derived from interconnected NCs.^29^Fig. 4 indicates 77.8 m^2^ g^−1^ as the specific surface area of A-TiO_2_ NCs. Furthermore, its narrow pore-size distribution centered at 13.0 nm was ascribed to the presence of relatively small inner pores between NCs and a large pore constructed by an independent structure, which agrees with the FE-SEM and HR-TEM analyses as well. Additionally, the standard multi-point BET technique was employed to calculated the specific surface area of 14.2 m^2^ g^−1^ for R-TiO_2_, which was only one-fifth that of A-TiO_2_. The narrow pore-size distribution exhibited in the as-prepared R-TiO_2_ was centered at 60.2 nm. Basically, both DSSCs and heterogeneous catalyst applications are porosity dependent due to the increase in the dye adsorption and active centers.

### DSSC performance

**Fig. 5:**
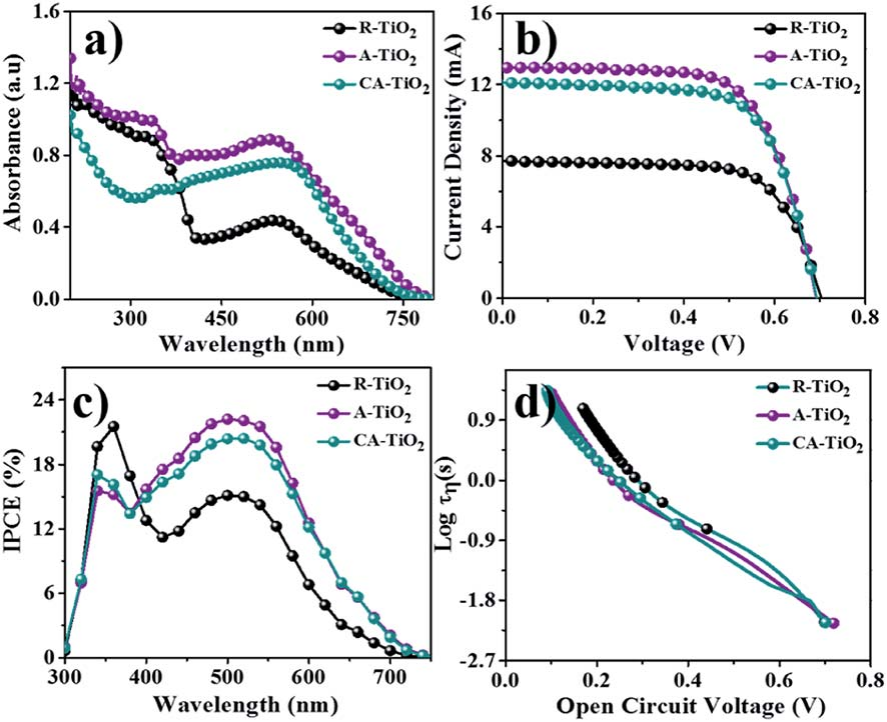
(a) UV-Vis, (b) *J*–*V*, (c) IPCE, and (d) *τ* (*vs. V*_oc_) measurements of R-, A- and CA-TiO_2_ photoanodes.

The DSSC performance of CA-TiO_2_ (∼20 nm particle-size, 100% anatase, ENB Korea) NCs was also measured as a reference. The performances of A- and CA-TiO_2_ NCs based DSSCs are given in Fig. 5. The UV-Vis absorption spectra of both photoanodes, with N-719 dye, were obtained in a wide visible wavelength range. The characteristic peak hump appeared at 530 nm was due to the N-719 dye (Fig. 5a). The N-719 dye adsorption over A-TiO_2_NCs (0.86 × 10^−7^ mol cm^−2^) was higher than those over CA-TiO_2_ (0.81 × 10^−7^ mol cm^−2^) and R-TiO_2_ NCs (0.42 × 10^−7^ mol cm^−2^), which possibly as a result of the higher surface area and narrow particle size of A-TiO_2_. To quantify the dye-loading amounts over two photoanodes, these sensitized photoanodes were immersed into 0.1 M sodium hydroxide solution to desorb the adsorbed dye molecules, followed by measuring the UV-Vis absorbance spectra. Using the Beer–Lambert law, the amounts of dye absorbed over these electrodes were determined and are listed in Table 1. The current density–voltage curves of both DSSCs, *i.e.*, CA-TiO_2_ and, A-TiO_2_ NCs photoanodes, under one sun illumination were measured and are plotted in Fig. 5b. Table 1 presents a summary of the DSSCs outcome based on various parameters for both photoanodes, where, in both cases, the open circuit voltage (*V*_OC_) of 0.70 V was nearly same because *V*_OC_ mainly is determined by the difference between the oxidation potential of the electrolyte and the Fermi level of the photoelectrode.^30^ Although, the as-obtained short-circuit current densities (*J*_SC_) of both DSSCs confirmed higher performance in A-TiO_2_ NCs photoanode compared to that in CA-TiO_2_, could be assigned to a narrow particle size as A-TiO_2_ demonstrates more dye molecules than that of CA-TiO_2_.^31^

**Table 1:** Summary of DSSCs average efficiency were obtained using six devices under AM 1.5G illumination (100 mW cm^−2^)

Photoanodes	*J*_SC_ (mA cm^−2^)	*V*_OC_ (V)	ff (%)	PCE (%)	Dye adsorption mol cm^−2^	Crystal size (nm)
R-TiO_2_	7.7	0.71	69	3.7	0.42 × 10^−7^	19.5
Std. dev.	0.089	0.023	0.018	0.019		
A-TiO_2_	12.9	0.70	67	6.0	0.86 × 10^−7^	12.0
Std. dev.	0.082	0.020	0.011	0.012		
CA-TiO_2_	12.0	0.70	65	5.8	0.81 × 10^−7^	16.2
Std. dev.	0.088	0.022	0.016	0.017		

The presence of an interconnected architecture of NCs in A-TiO_2_ electrode could provide a long-range charge-transfer channels, resulting in 12.9 mA cm^−2^*J*_SC_, 0.70 V *V*_OC_, 67% ff, and 6.0% PEC, which were considerably higher than those of CA-TiO_2_ (12.0 mA cm^−2^*J*_SC_, 0.70 V *V*_OC_, 65% ff, and 5.8% PEC) and R-TiO_2_ electrodes (7.7 mA cm^−2^*J*_SC_, 0.71 V *V*_OC_, 69% ff, and 3.7% PEC), Fig. 5. The significant enhancement in the DSSCs performance of A-TiO_2_NCs photoanode could be due to its unique morphology, high electrical conductivity (2.96 × 10^−7^ Ω cm), relatively high specific surface area (77.8 m^2^ g^−1^), and optimum electron mobility (0.124 cm^2^ V^−1^ s^−1^). Fig. 5c reveals the incident photon-to-electron conversion efficiency (IPCE) spectra of R-TiO_2_, A-TiO_2_, and CA-TiO_2_ photoanodes, where two peaks, one at around 350 nm (TiO_2_) and the other at around 520 nm, indicative of the absorbed N-719 dye, were observed.^32^ The as-synthesized A-TiO_2_ photoanode confirmed a maximum IPCE of 22.08%, which was 20.31% in CA-TiO_2_, and 15.12% in R-TiO_2_ photoanodes, revealing a higher N-719 dye adoption of A-TiO_2_ over CA-TiO_2_ and R-TiO_2_. The open-circuit voltage decay plots of both electrodes measured under applying open circuit voltage conditions with respect to time are shown in Fig. 5d. At zero current, there was an exponential decrease in *V*_OC_, which supports the recombination of electrons.^33^ In the graph, the A-TiO_2_ photoanode exhibited a slow decrement in *V*_OC_, which is due to the electron recombination mitigation. The calculated lifetime of the electron, *i.e.*, the time taken by an excess minority carrier to recombine, of A-TiO_2_, determined by the *V*_OC_ decay method, was higher than those of the others, suggesting that the electrons are more likely to reach the working electrode than CA-TiO_2_ and R-TiO_2_ photoanodes.^34^

**Fig. 6:**
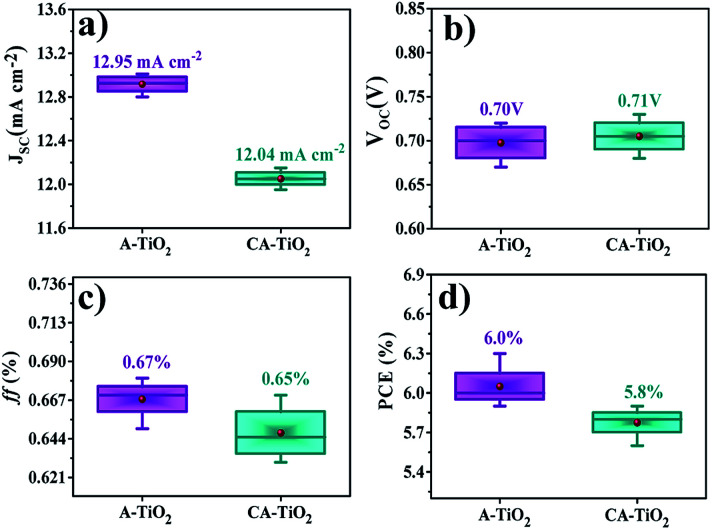
Summary of average performance of six devices: (a) *J*_sc_, (b) *V*_oc_, (c) ff, and (d) PCE of CA- and A-TiO_2_-based. The maximum/minimum and average values are denoted by — and ●, respectively.

Fig. 6a–d summarizes the average performances of CA-TiO_2_ and A-TiO_2_ photovoltaic devices. The as-synthesized A-TiO_2_ photoanode exhibited a high reproducibility of *J*_SC_, *V*_OC_, ff, and PEC with a comparatively small standard deviation of 1.72%, which is substantially higher than those for the CA-TiO_2_ photoanode-based DSSCs devices (1.20%), suggesting the possibility of its reusability compared to the case with others.

### Energy levels

**Fig. 7:**
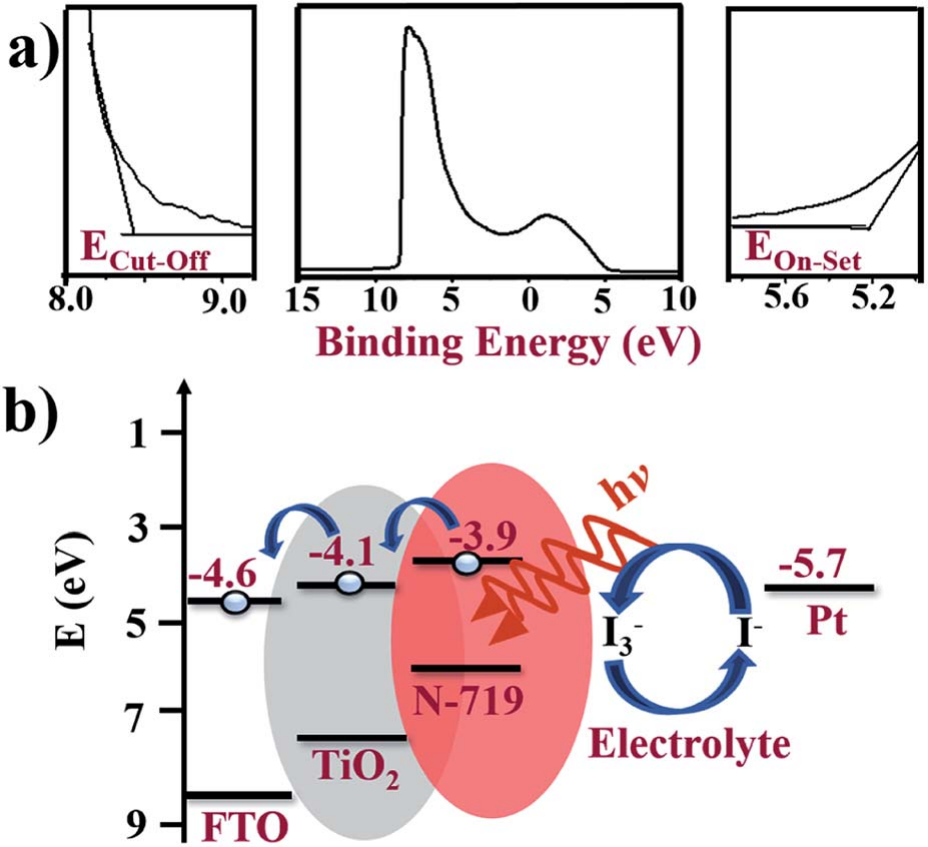
(a) UPS spectra shown three different energy edge ranges: cut-off region, survey binding energy spectra, and onset region of A-TiO_2_. (b) The schematic energy bandgap diagram of FTO/A-TiO_2_/N-719 dye/electrolyte/Pt photoanode device calculated from UPS analyses.

To confirm the energy alignment of dye and TiO_2_, UPS measurement was performed, which is shown in Fig. 7a, where the various energy edge ranges such as the cut-off region, survey binding energy spectra, and onset region for A-TiO_2_ NCs are clearly indicated. A schematic energy band diagram of the DSSCs based on the A-TiO_2_ NCs photoanode charge transfer process is shown in Fig. 7b. The high binding energy cut-off (*E*_cut-off_) of the A-TiO_2_ NCs photoanode was 8.56 eV. The binding energy onset (*E*_onset_) was −5.30 eV, relative to the Fermi level of Au (at 0 eV). The valence band minimum (VBM) calculated using VBM = *hν* − (*E*_cut-off_ − *E*_onset_) equation, whereas, *ν* is the frequency of vibration of light and *h* is the Planck's constant estimates VBM of −7.35 eV for A-TiO_2_ NCs. The band gap energy denoted by (*E*_g_) of the A-TiO_2_ NCs electrode was estimated using the following equation:

1 (*αhν*)^2^ = *A*(*hν* − *E*_g_),

where, *α* is the absorption coefficient, and *A* is the proportionality constant. Fig. S4† presents a UV-Vis spectrum of the A-TiO_2_ photoanode, where 3.2 eV *E*_g_ was obtained for A-TiO_2_NCs film, which closely matches to the reported value.^35^ Using this band gap energy, the conduction band minimum (CBM) of A-TiO_2_ was calculated using the following equation:

2 CBM = VBM + *E*_g_.

The calculated CBM of the A-TiO_2_ NCs electrode was −4.1 eV. To understand the electron transfer pathways of this photoanode, the energy band diagram of the FTO/A-TiO_2_/N-719 dye/electrolyte/Pt DSSCs was also designed. In DSSCs, on capturing photons over the N-719 dye layer, the electron–hole pairs are created, and the excited electrons are injected into the CBM of the A-TiO_2_NCs, which are then collected at the FTO substrate of the −4.6 eV work function more easily.^36^

### Electrochemical charge transfer process

**Fig. 8:**
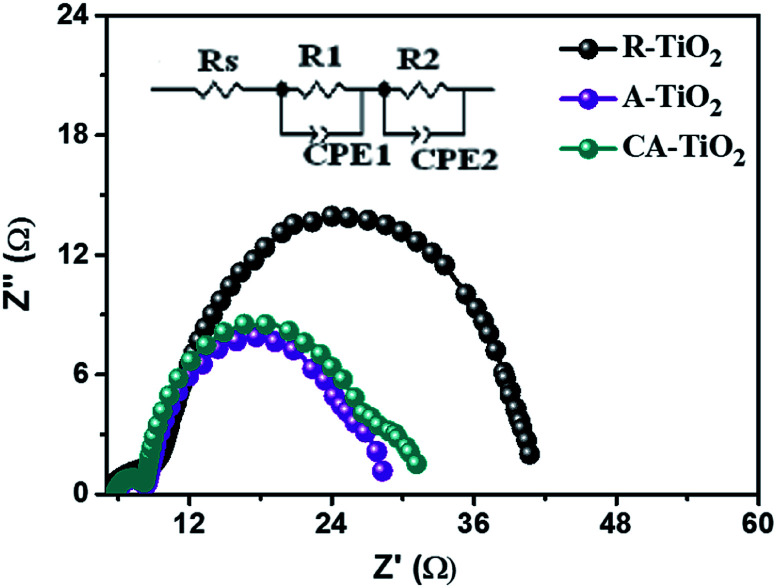
EIS plot of the R-, A- and CA-TiO_2_ NCs photoanodes.

The photogenerated charge transfer and recombination processes are crucial in DSSCs performance, which can be explained with EIS measurement operated bias potential 0.70 V under dark condition. Fig. 8 shows the Nyquist curve of the A-TiO_2_ NCs photoanode which demonstrates one semicircle at high-frequency region (>1 kHz), and another semicircle at a low-frequency region (100–0.1 Hz). A relatively small semicircle at high-frequency region corresponds to the charge-transfer resistance (*R*_1_), and the capacitance (CPE_1_) at the counter electrode (platinum)/redox electrolyte interface. The second semicircle, at the low-frequency range, corresponds to the charge-transfer interactions occurring at the photoanode-dye/electrolyte interface (*R*_2_), and the chemical capacitance (CPE_2_) at the TiO_2_/dye/redox electrolyte interface.^37^ The equivalent circuit and the corresponding values obtained by the Z view software are shown as an inset of Fig. 8, and also in Table 2.

**Table 2:** The R-TiO_2_ and A-TiO_2_ NCs photoanodes fitted electronic parameters of Nyquist plots

	RS	R1	R2	CPE1-T	CPE1-P	CPE2-T	CPE2-P	*τ* (ms)
R-TiO_2_	5.2	4.4	31.0	5.32 ×10^−3^	0.59	7.90 ×10^−3^	0.91	1.58 × 10^−4^
A-TiO_2_	5.9	2.4	19.7	1.06 ×10^−3^	0.73	21.0 ×10^−3^	0.87	2.24 × 10^−4^

### Effects of the catalyst amount, temperature, time, and solvent system

**Scheme 3:**
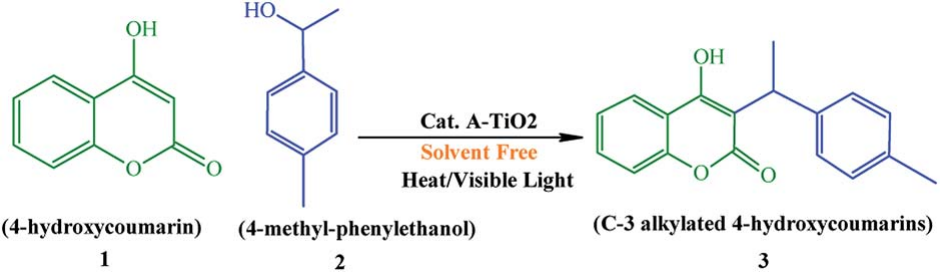
Synthesis of the substituted C-3 alkylated 4-hydroxycoumarins using the A-TiO_2_ catalyst.

For the synthesis of C-3 alkylated 4-hydroxycoumarins, we successfully analyzed the reaction conditions, for instance catalyst amount, temperature, and required time for the completion of the reaction (Scheme 3, Table 3). In first, of A-TiO_2_ catalyst amount was tested; among them a model amount of 20 mg exhibited a notable yield. Although, when the catalyst amount was increased to 30 mg, no obvious change in the yield was observed, this may be due to the exhaustion of the catalytic sites or the maximum conversion of the catalyst efficiency.

**Table 3:** The optimization of reaction conditions determines

No	Amount of catalyst (mg)	Temperature (°C)	Solvent	Time (h)	Yield^b^ (%)
1	0	Reflux	MeCN	10	—
2	5	Reflux	MeCN	1	0
3	10	50 °C	MeCN	1	0
4	15	50 °C	MeOH	1	0
5	20	50 °C	EtOH	1	0
6	20	50 °C	THF	1	0
7	20	50 °C	DCM	1	0
8	20	50 °C	IPA	1	0
9	20	50 °C	Toluene	1	0
10	20	50 °C	Solvent free	5	75
11^a^	20	70 °C	Solvent free	5	95
12	30	70 °C	Solvent free	5	95
13	20	Visible light	Solvent free	5	95

a Reaction conditions: 4-hydroxycoumarin (1 mmol), 4-methylphenylethanol (1 mmol), catalyst (20 mg), solvent free.

b Isolated yields.

Under the optimized experimental conditions, we then performed the reactions with different solvents and varying reaction conditions (reflux, 50 °C, 70 °C, and under visible light irradiation) for different time periods (1–10 h). In the presence of 20 mg of A-TiO_2_ catalyst, at 70 °C for a reaction time of 4.5 h, 95% yield was obtained, which is one of the maximum values, so far, reported in the literature.^38–47^ It was observed that after extended reaction time and high temperature, the reaction did not proceed in the forward direction. In our study, 20 mg of A-TiO_2_ catalyst under heat/visible light and solvent free conditions demonstrated a notable product yield of 95% with stipulated time period of 4.5 h. Similarly, the nature of the solvent can interrupt the reaction environment by affecting the catalytic stability, solubility, and rate of the reaction. After careful examination of all the results, a solvent-free condition was preferred for the synthesis of further derivatives. Thereafter, this solvent-free specific condition was used for all the reactions. Table 3 summarizes the percentage yield of C-3 alkylated 4-hydroxycoumarins under distinct reaction condition *i.e.* catalyst amount, reaction temperature, various solvents, and time.

### Synthesis of substituted C-3 alkylated 4-hydroxycoumarins using the A-TiO_2_ catalyst

Under the optimal re substituted C-3 alkylated 4-hydroxycoumarins using the A-TiO_2_ catalyst (Table 4).

**Table 4:** C-alkylation of 4-hydroxycoumarin with various alcohols

No	Alcohol	Product	Time (h)	Yield %
1	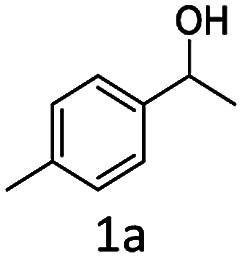	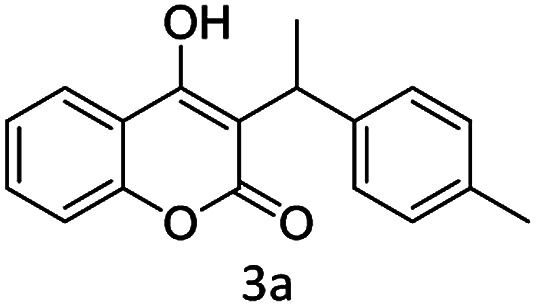	4.5	97
2	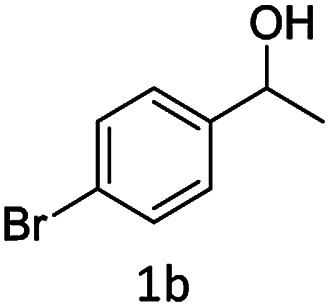	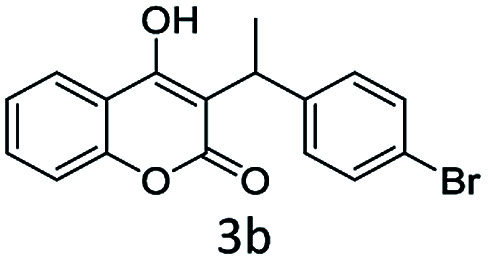	5	95
3	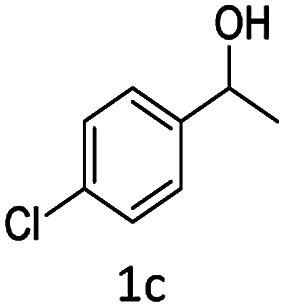	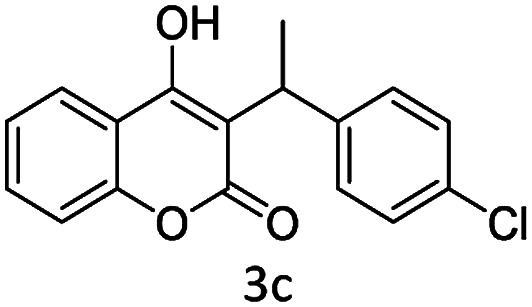	5	94
4	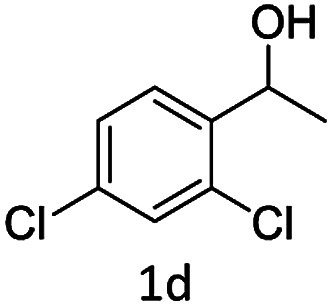	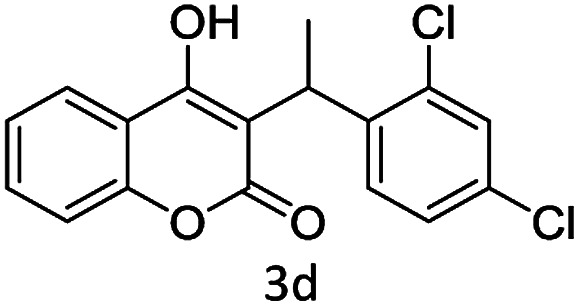	5	90

### The plausible reaction mechanism

**Scheme 4:**
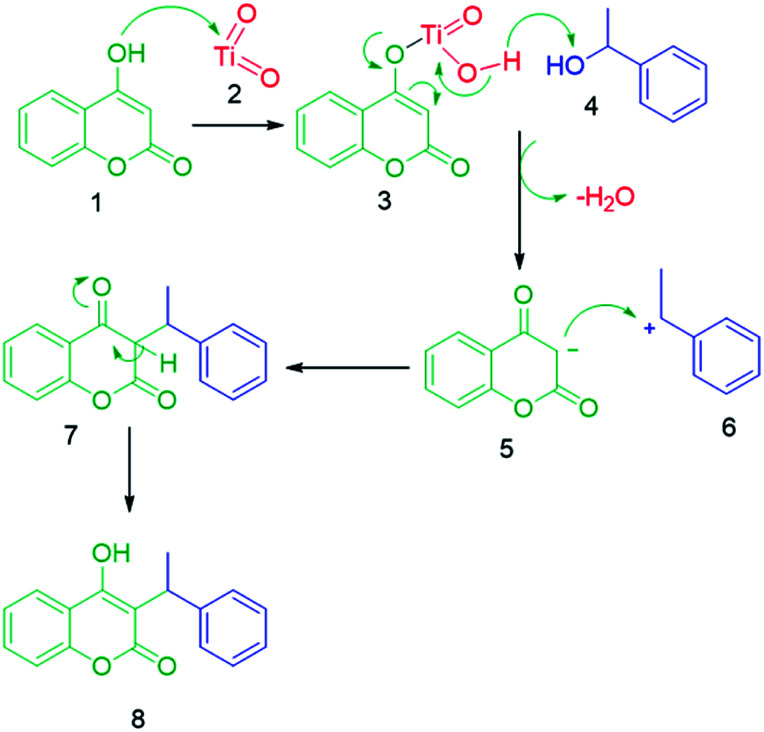
Plausible reaction mechanism for the synthesis of C-3 alkylated 4-hydroxycoumarins.

A-TiO_2_-catalyzed synthesis of C-3 alkylated 4-hydroxycoumarins occurred by removal of a water molecule, which results in the formation of a stable carbocation, as alkylating species that were derived from alcohol on the Lewis acidic sites of A-TiO_2_ (C-3 alkylation). The enolic hydroxy group of 4-hydroxycoumarin was activated by Ti metal *via* Lewis acid catalysis to increase the nucleophilicity of the 3-position of 4-hydroxycoumarin. The final product C-3 alkylated 4-hydroxycoumarins was produced through an electrophilic attack activated secondary benzyl alcohols on the nucleophilic enolic hydroxy group of 4-hydroxycoumarin (Scheme 4).

### Recyclability of the A-TiO_2_ nanocatalyst

After evaluating the activity and flexibility of the A-TiO_2_ catalyst for different kinds of reactions, the recyclability of the catalyst was observed using the reaction between 4-hydroxycoumarin and 4-methyl-phenylethanol as a model reaction under the optimal conditions. Typically, after completion of reaction, the A-TiO_2_ catalyst was separated from the reaction mixture through a simple filtration method. The reusability of the catalyst was assessed after activating the catalyst at 200 °C for 1 h; the catalyst was employed for five runs to test the consistency of its activity. After five runs, the product yields obtained over the A-TiO_2_ catalyst exhibited no noticeable decrease, suggesting a high consistency of the activity of the as-synthesized A-TiO_2_ catalyst. This reusability study reveals the high stability and turnover of A-TiO_2_ nanocatalysts under optimal operating conditions. The reusability of the catalysts is one and significant advantage that makes them beneficial for marketable applications.

## Conclusions

In summary, A-TiO_2_ was successfully prepared *via* a simple, elegant and cost-effective hydrothermal method in the presence of d-mannitol. In this synthesis, d-mannitol plays a crucial role in the fabrication of A-TiO_2_ NCs, as a; (a) complexing agent that regulates the zigzag chains of the TiO_6_^2−^ octahedral by dominant edge sharing, and (b) capping agent that influences the nucleation for NCs during the hydrolysis process. The FTIR spectrum impeccably suggests the formation of an intermediate complex structure between Ti^4+^ and d-mannitol ions. A PCE of 6.0% is achieved from A-TiO_2_ NCs photoanode, which is about 1.6 times higher than R-TiO_2_ (3.7%); this may be due to the difference in the structure, surface area, dye intake capacity, and charge transfer resistance. In addition, A-TiO_2_ catalyst was used for synthesizing of pharmaceutically significant C-3 alkylated 4-hydroxycoumarins under solvent-free conditions. An excellent yield of 95% at very less reaction time of 4.5 h is obtained. The all synthesized compounds are confirmed by ^1^H NMR spectroscopy. The catalytic activity of produced A-TiO_2_ has remarkable recyclability up to five consecutive runs with slight change in formed product yields, which clearly demonstrates the novel, efficient, recyclable, and economical nature of the as synthesized A-TiO_2_ catalyst. Therefore, the A-TiO_2_ catalyst can be a potential candidate, over other expensive and rare-earth catalysts, during organic transformation processes.

## Conflicts of interest

There are no conflicts to declare.

## Supplementary Material

RA-010-D0RA01366H-s001
