# Supplementary material for: Phase controlled synthesis of bifunctional TiO2 nanocrystallites viad-mannitol for dye-sensitized solar cells and heterogeneous catalysis
Source: RSC Adv. 2020 Apr 14;10(25):14826–36. doi: 10.1039/d0ra01366h (PMC9052067; doi:10.1039/d0ra01366h)
Supplement: RA-010-D0RA01366H-s001 [file RA-010-D0RA01366H-s001.pdf]

Electronic Supplementary Material (ESI) for RSC Advances.
This journal is © The Royal Society of Chemistry 2020

Supporting Information (SI)

Phase Controlled Synthesis of Bifunctional TiO_2 Nanocrystallites via D-mannitol for Dye-Sensitized Solar Cells and Heterogeneous Catalysis

Abdullah M. Al-Enizi[a], *T. A. J. Siddiqui*[b], *Shoyebmohamad F. Shaikh*[a], *Mohd Ubaidullah*[a*], *Ayman Yousef*[c], *Rajaram S. Mane*[b]*, *and Abu ul Hassan Sarwar Rana*[d]*

[a]Department of Chemistry, College of Science, King Saud University, Riyadh 11451, Saudi Arabia

[b]School of Physical Sciences, Swami Ramanand Teerth Marathwada University, Nanded-431606, M.S., India

[c]Department of Mathematics and Physics Engineering, Faculty of Engineering at Mataria, Helwan University, Cairo 11718, Egypt.

[d]Department of Intelligent Mechatronics Engineering, Sejong University, Seoul, South Korea.

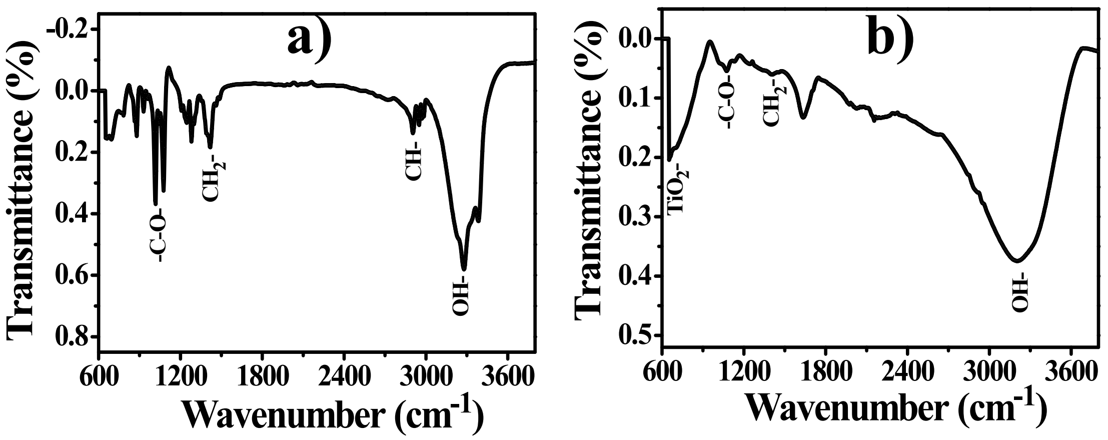

Fig. S1 FTIR spectra of; a) pure D-mannitol, and b) an intermediate complex of TiO_2–D-mannitol.

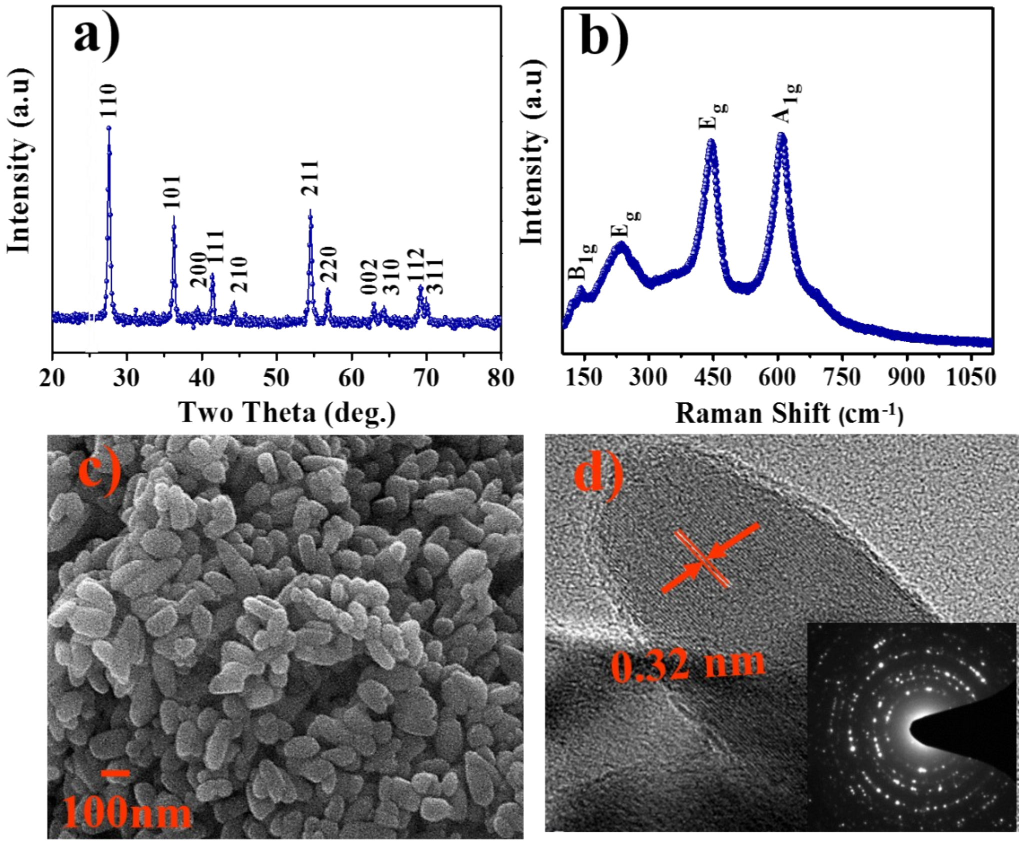

Fig. S2 a) X-ray diffraction pattern, b) Raman analysis, c) FE-SEM images, and HR-TEM images of R-TiO_2 was obtained without assistance of D-mannitol.

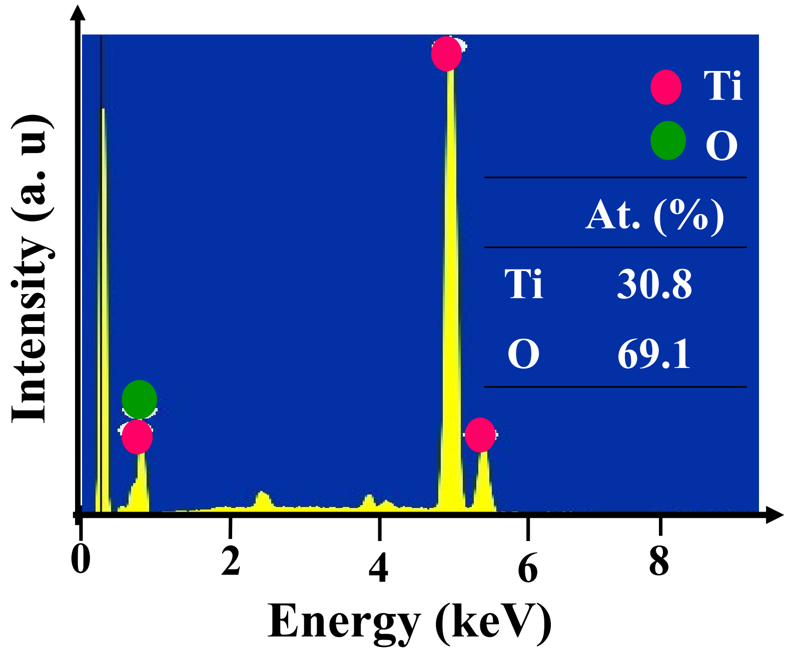

Fig. S3 the elemental stoichiometry determined by EDX mapping of TiO_2.

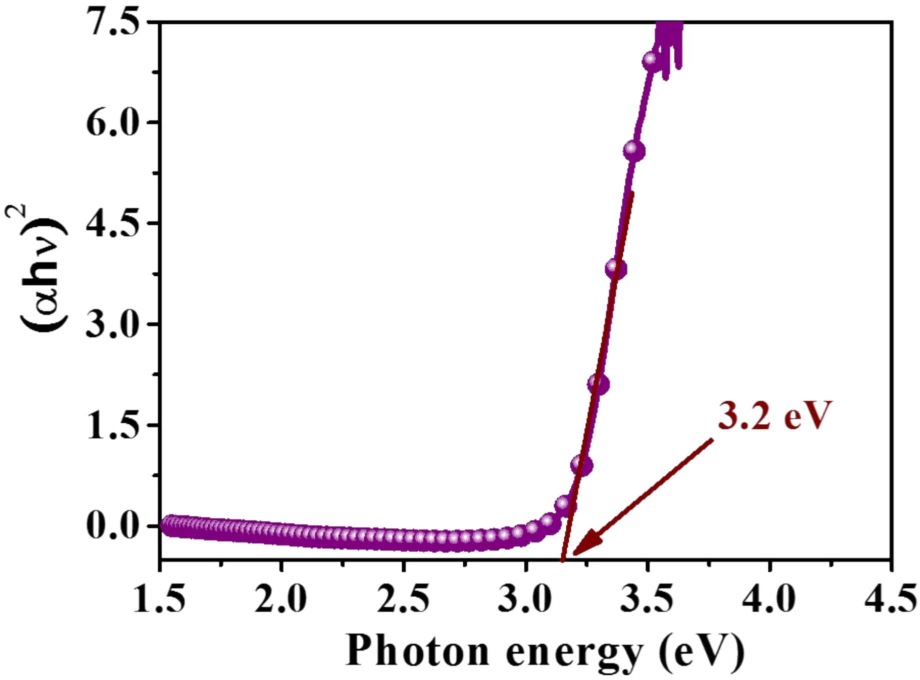

Fig. S4 Uv-Vis band gap of A-TiO_2 photoanode measured on FTO substrate.

1) **^{1}HNMR of compound 3a**

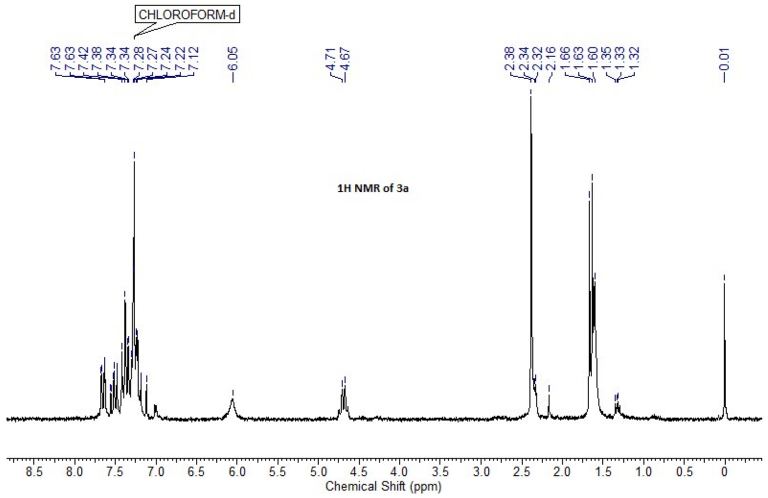

2) **^{1}HNMR of compound 3b**

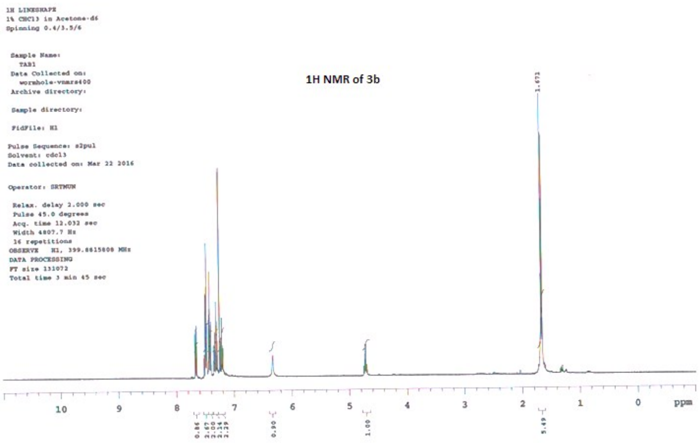

3) ^{1}HNMR of compound 3c

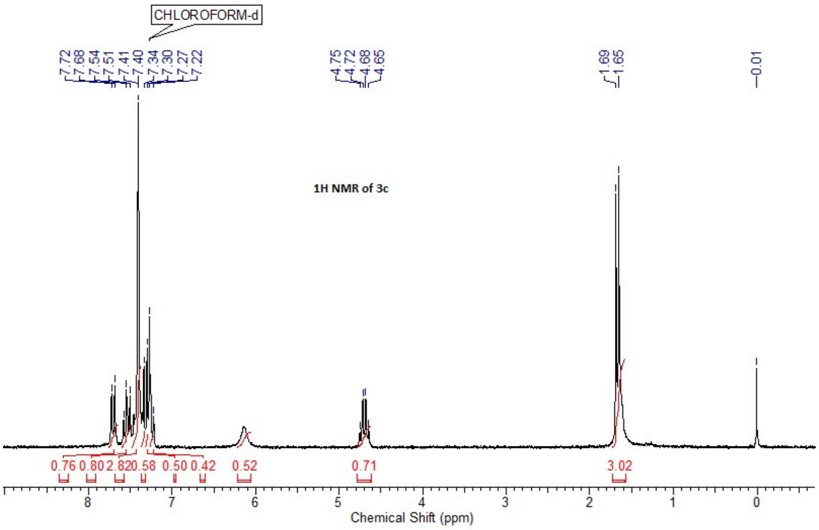

4) 1HNMR of compound 3d

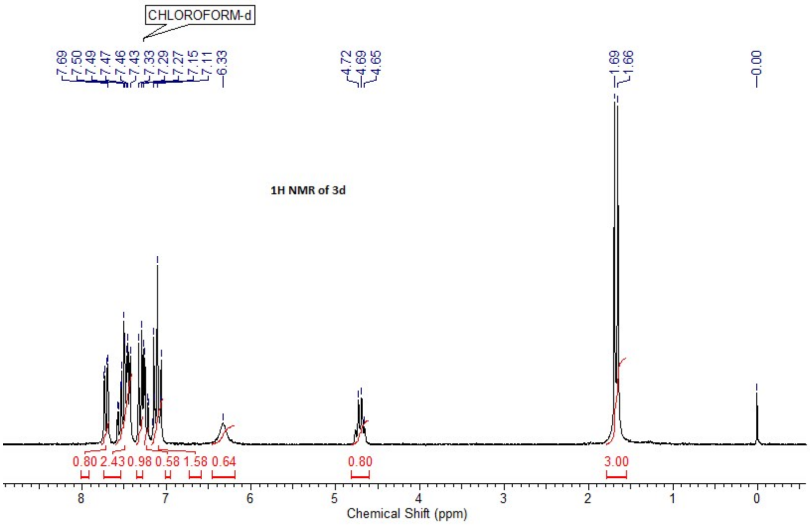

Table S1: Hall Measurement and electrical properties of A-TiO_2 nanocrystallite thin film on glass substrate.

Photoanode	Resistivity $\times 10^6$ Ω cm	Conductivity $\times 10^{-7}$ / Ω cm	Mobility cm^2/Vs	Sheet Concentration $\times 10^9$ $/cm^2$
A-TiO_2	3.35	2.96	0.124	-2.38
